# Combined Herbal Eye Drops Exhibit Neuroprotective and Intraocular Pressure-Reducing Effects in a Glaucoma Rat Model

**DOI:** 10.3390/antiox14050549

**Published:** 2025-05-01

**Authors:** Tibor Rak, Evelin Patko, Edina Szabo, Alexandra Vaczy, Dorottya Molitor, Dora Reglodi, Adrienne Csutak, Tamas Atlasz

**Affiliations:** 1 Department of Ophthalmology, Medical School—Clinical Centre, University of Pécs, 7624 Pécs, Hungary; rak.tibor@pte.hu (T.R.); csutak.adrienne@pte.hu (A.C.); 2 Department of Pharmacognosy, Faculty of Pharmacy, University of Pécs, 7624 Pécs, Hungary; 3 Department of Anatomy, HUN-REN-PTE PACAP Research Team, Centre for Neuroscience, Medical School, University of Pécs, 7624 Pécs, Hungary; evelin.patko@gmail.com (E.P.); edina.szabo@aok.pte.hu (E.S.); alexandra.vaczy@aok.pte.hu (A.V.); modxaat.pte@tr.pte.hu (D.M.); dora.reglodi@aok.pte.hu (D.R.); 4 Department of Sports Biology and Kinesiology, Faculty of Sciences, University of Pécs, 7624 Pécs, Hungary

**Keywords:** glaucoma, herbal, eye drops, neuroprotection, OCT, ERG

## Abstract

(1) Background: Glaucoma is a multifactorial group of diseases characterized by progressive optic neuropathy. Intraocular pressure (IOP) is the only successfully modifiable risk factor for all forms of glaucoma. However, recent research has highlighted the reduction of oxidative stress and neuroinflammation as promising therapeutic targets. In this study, we evaluated the antiglaucomatous effects of a combined herbal extract applied as eye drops in a rat model of glaucoma. (2) Methods: Sprague Dawley rats were divided into four groups: healthy controls, glaucomatous animals treated with preservative-free artificial tears, and healthy and glaucomatous groups receiving combined herbal-based eye drops for 8 weeks. Glaucoma was induced through injection of microbeads into the anterior chamber at week 1 and week 3. Before the first injection and at weeks 4 and 8, rats underwent optical coherence tomography (OCT) and electroretinogram (ERG) recordings. Retinal analyses were conducted to assess retinal ganglion cell (RGC) count, vessel density, and markers of neural pathways, oxidative stress, and inflammation. (3) Results: The combination of herbal extracts showed beneficial effects on IOP elevation, and significantly improved ERG responses. Neuroprotective effects were assessed using OCT, immunohistochemistry, and proteomics. Most parameters in herbal eye drop-treated rats were not statistically different from those in healthy controls. (4) Conclusions: Topical administration of plant-based compounds may serve as an effective supportive therapy for ocular hypertension and retinal neuroprotection.

## 1. Introduction

Glaucoma is a neurodegenerative disease and the leading cause of irreversible vision loss worldwide [1,2,3]. It is primarily caused by the slow destruction of optic nerve fibers, with primary open-angle glaucoma (POAG) accounting for over 70% of cases [1,4]. The disease has no cure, but early detection and treatment can slow its progression and help preserve vision. The incidence is increasing, with glaucoma becoming the second leading cause of vision loss after cataracts in 2022 [1,2,3]. By 2040, glaucoma is expected to affect approximately 112 million people between the ages of 40 and 80 [1,2,3,4]. Glaucoma is associated with an inflammatory response mediated by increased pro-inflammatory cytokines [3,5] and harmful free radical formation [3,6] in the lamina cribrosa, retina, anterior chamber angle structures, and aqueous humor [3,7]. Current treatment procedures primarily aim to reduce intraocular pressure (IOP) through pharmacological, laser, and surgical interventions [2,3]. Neuroprotection has become an essential aspect of complementary glaucoma therapies, aiming to slow down apoptosis and functional deterioration in retinal ganglion cells [2,3,8]. In addition to conventional treatments, complementary therapies, such as herbal remedies, dietary modifications, and nutritional supplements, are increasingly used by glaucoma patients [8]. A Canadian survey found that 13.6% of glaucoma patients used these alternative methods, frequently without disclosing this to their healthcare providers [8,9,10]. The adoption of these therapies correlates with a younger age at diagnosis, higher educational attainment, and the use of multiple treatment modalities [8,9,10]. Despite extensive research on the potential benefits of various supplements, the evidence remains inconclusive, and these complementary therapies have yet to meet evidence-based standards.

The experiments reported here are based on our developed herbal combination (Application No: P2500037). The selected herbal components for our combined formulation were chosen based on their chemical constituents (Table 1) and therapeutic effects, as documented in the ophthalmological literature. *Rosmarinus officinalis L.* (Lamiaceae), a widely used culinary and medicinal herb in European and Hungarian folk medicine, exhibits notable neuroprotective and anti-inflammatory properties, primarily attributed to rosmarinic acid (Table 1). Rosmarinic acid modulates pathways such as VEGF (vascular endothelial growth factor) and AChE (acetylcholinesterase) inhibitory pathways [11,12,13], potentially preventing hypoxia-induced angiogenesis and related complications in conditions like glaucoma. Besides VEGF, rosmarinic acid inhibits several proapoptotic and proinflammatory factors, such as TGFβ (transforming growth factor beta), IL(interleukin)-6, and TNFα (tumor necrosis factor alpha), partly through the NFκB (nuclear factor kappa B)/p65 pathways [11,12,13]. In ophthalmological research, rosmarinic acid has been used as an anti-angiogenic and anti-fibrotic agent in experimental filtration glaucoma surgery (trabeculectomy) in rabbits, as well as in intravitreal implants [13,14]. However, no studies have investigated its IOP-lowering effects or its impact on glaucoma progression in glaucomatous animal models. Its safety profile has been validated in animal studies, showing no retinal toxicity even at high doses [11,12,13,14]. Other bioactive compounds, including carnosic acid and luteolin [15] (Table 1), further contribute to its antioxidant and therapeutic effects. As an EU-registered food additive (E392), rosemary extracts are widely available and cost-effective [16]. *Foeniculum vulgare Mill.* (Apiaceae) is another non-toxic herb known for its use in teas, confections, and spices. Studies suggest its aqueous extracts reduce IOP in a single glaucomatous animal model, likely due to acetylcholinesterase inhibition [17]. The primary active compound, trans-anethole (Table 1), demonstrates anti-inflammatory, and antioxidant (aldose reductase inhibitory) activities [17,18,19]. Among organic acids, rosmarinic acid is also present in significant amounts in the herb (Table 1). Other studies in rodents have described the herb’s neuroprotective effects, mediated through the inhibition of microglial activity and activation of astrocytes, as well as through effects on AChE and γ-aminobutyric acid-ergic (GABAergic) activity [18,19], though its effects on ocular neuroprotection remain underexplored. *Helichrysum italicum* (Roth) *G. Don fil.* (Asteraceae) contains anti-inflammatory and antioxidant flavonoids such as rutoside and chlorogenic acid (Table 1), which inhibit pathways like the NFκB, IL-1β, TNFα, IL-6, IL-8, and prostaglandin-E2 and promote tissue repair [20]. This also affects the inflammatory 5-lipoxygenase (5-LO) and cyclooxygenase (COX-1 and COX-2) pathways [20]. Although its potential for neuroprotection in glaucoma is hypothesized based on these properties, *H. italicum* has not traditionally been used in folk medicine for treating glaucoma or other eye diseases [20,21,22]. Additionally, no studies have yet evaluated its effects on ocular health.

These three herbs, naturally abundant in Central Europe, are cost-efficient, safe, and compliant with the *Pharmacopoeia Hungarica (Ed. VIII.)* and *Europea (Ed. X.)* [43]. Formulating their extracts into daily-use eye drops could offer neuroprotection and help reduce IOP in glaucoma patients. Preliminary studies suggest that individual compounds may be effective, but their combined potential in glaucoma therapy remains unknown. This novel formulation aims to address this gap by leveraging the broad safety margins and accessibility of these herbs for therapeutic use. Our research focuses on developing a novel herbal-based therapeutic formulation with anti-inflammatory and antioxidant properties. The goal is to reduce IOP and slow the progression of irreversible optic neuropathy. This study highlights the potential of natural therapies as cost-effective adjuncts in managing retinal diseases.

## 2. Materials and Methods

### 2.1. Animals

All animal procedures were approved by the Institutional Animal Welfare Committee of the University of Pécs (PTE-MÁB) and authorized by the Baranya County Government Office (permit number: BA02/2000-68/2022; animal experiment reference number: KA-3637), in accordance with “XXVIII 1998 Act on the protection and welfare of animals”. Male Sprague Dawley rats (300–500 g and 2 months old) were obtained from Charles River Laboratories (USA) and housed in the animal facility of the Department of Anatomy, University of Pécs (permit number: BAHU0104L07). Rats were kept in type III H cages (2 animals per cage; minimum area: 450 cm^2^/animal) under controlled conditions: temperature 20–24 °C, humidity 50–60%, and a 12/12 h light/dark cycle. They were fed and given water ad libitum. Before the experiments, animals were divided into four groups: (1) placebo-treated control (PBS + P; *n* = 11), (2) herbal treatment control (PBS + H; *n* = 6), (3) placebo-treated glaucoma model (Bead + P; *n* = 21), and (4) herbal treatment glaucoma model (Bead + H; *n* = 15). Group sizes were based on our previous study (Szabo et al. [44]) and G-power *a priori* analysis (G*Power Version 3.1, Kiel, Germany), with a total of 57 animals included in the experiment. Four animals were utilized to conduct Draize tests with herbal active ingredients, both individually and in combination, prior to the formation of the groups. After invasive and non-invasive examinations, rats were monitored until they regained consciousness.

### 2.2. Rat Model of Glaucoma

Rats were anesthetized with an intraperitoneal injection of ketamine (90 mg/kg; Calypsol, Richter Gedeon, Budapest, Hungary) and xylazine (10 mg/kg; Sedaxylan, Dechra, Nortwich, United Kingdom, as according to the protocol of Sappington et al. [45]. To prevent infection, the ocular surface and surrounding tissues were cleaned with Braunol solution (B. Braun Medical AG, Melsungen, Germany). Glaucoma was induced in both eyes of the rats in Groups 3 (*n* = 21) and 4 (*n* = 15) by injecting 10 µL of fluorescent (580/603 nm) polystyrene microbeads (3.6 × 10^6^ beads/mL; FluoSpheres™, Thermo Fisher Scientific, Waltham, MA, USA) into the anterior chamber of each eye using a 33G Hamilton syringe. After the injections anti-inflammatory eye drops (Tobrex, 3 mg/mL; Alcon, Budapest, Hungary) were used to prevent inflammation and support corneal healing. In contrast, the rats in Groups 1 (*n* = 11) and 2 (*n* = 6) received 10 µL injection of phosphate-buffered saline (PBS) into both eyes. The injection procedure was repeated after two weeks, based on the protocol described by Szabo et al. [44]. This two-step microsphere injection method ensured sustained IOP elevation for up to eight weeks, as observed in previous studies.

### 2.3. Topical Administration of Botanicals

Three botanicals—*Rosmarinus officinalis* (rosemary), *Foeniculum vulgare* (fennel), and *Helichrysum italicum* (immortelle)—with distinct mechanisms of action but potentially synergistic therapeutic effects were selected based on their pharmacological profiles and proven efficacy in the literature [3,8]. The eye drops were administered to both eyes of the rats, starting the day after the first microbead injection. Drops were applied twice daily: between 7:00 and 9:00 a.m. and 5:00 and 7:00 p.m. The placebo-treated groups (a portion of both the control and glaucoma groups, n = 32) received Hyabak^®^ (10 mL, Laboratoires Théa, Clermont-Ferrand, France) artificial tears containing a 1% sodium hyaluronate solution. The remaining animals in the control and glaucoma groups (*n* = 21) that received active treatment were administered a combined formulation of herbal active compounds (0.5% each), resulting in a total concentration of 1.5%, dissolved in a 1% sodium hyaluronate-based artificial tear solution. In the combined formulation, the primary active ingredient is 0.5% rosmarinic acid, derived from *R. officinalis* (≥98% (HPLC), Sigma–Aldrich, St. Louis, MO, USA), mixed with 0.5% diluent produced from the essential oil of *F. vulgare* [Diluendum foeniculi sine alcoholo FoNo (Adrienne Feller Cosmetics, Jászfényszaru, Hungary)] and 0.5% commercially available hydrolate of *H. italicum* (Adrienne Feller Cosmetics, Jászfényszaru, Hungary). This formulation not only incorporates natural antioxidants to support ocular health but also enhances stability and shelf life through its biochemical processes. In addition to its accessibility, the selection of hyaluronic acid-containing artificial tears as the vehicle was based on pharmacological research findings. Hyaluronic acid-containing artificial tears not only form a protective film on the ocular surface [46] but are also considered safe for counteracting the adverse effects of elevated IOP on the ocular surface [46], particularly in patients with dry eye disease and glaucoma.

### 2.4. Measurement of IOP

IOP was noninvasively measured weekly in all rats (*n* = 53) in both eyes with a rebound tonometer (Tonolab, iCare, Vantaa, Finland) to test the efficacy of the combined herbal eye drops. Multiple sampling procedures (6 measurements) were performed once a week between 7 and 9 a.m.

### 2.5. Electroretinography (ERG)

ERG was used to monitor retinal function in glaucoma, as it provides information on the activity of photoreceptors, bipolar, and amacrine cells [44,47]. Previous studies have shown that functional changes in the retina can be detected by ERG before ganglion cell apoptosis occurs [48]. Before starting the experiment, functional analysis (ERG) was performed on each animal (*n* = 40) to establish baseline vision function. Scotopic ERG measurements were conducted on both eyes at baseline (day 0) and repeated at 4 weeks and 8 weeks post-treatment. Animals were dark-adapted for at least 12 h before the test and anesthetized with intraperitoneal ketamine (90 mg/kg) and xylazine (10 mg/kg) cocktails. Each rat was placed on a heating pad throughout the experiment, and their pupils were dilated by one drop of 1% homatropine (*w*/*v*, Humapent-Teva, Debrecen, Hungary). ERG recordings were conducted with three electrodes. The reference electrode was placed subcutaneously between the eyes, the ground electrode was inserted under the ramp skin, and surface electrodes applied on the central cornea transduced the electronic potential from the retina responding to light stimuli. Light stimuli (50 pulses at 5 cd s/m^2^, 0.25 Hz, 503 nm LED light) were used, and signals were amplified (2000×, Bioamp SbA4-V6, Supertech, Pecs, Hungary) and recorded with an A/D converter (Ratsoft-Solar Electronic, Pecs, Hungary). ERG data were averaged (*n* = 50/eye) and analyzed for a-wave and b-wave amplitudes using OriginPro 2018 (OriginLab Corporation, Northampton, MA, USA) software. The following parameters were observed: amplitude and implicit time of a-waves, b-waves, and oscillatory potential (OP).

### 2.6. Optical Coherence Tomography (OCT)

The retinal structure, composed of ten distinct layers, was imaged using spectral domain optical coherence tomography (SD-OCT) (Bioptigen, Research Triangle Park, NC, USA) for high-resolution, real-time imaging of the anterior and posterior segments of the eye [49]. OCT imaging was first performed on day 0 (pre-treatment) after ERG measurements, while the animals (n = 41) were still anesthetized and had dilated pupils. Preservative-free artificial tears (Systane^®^, Alcon, Budapest, Hungary) were applied to protect and hydrate the corneal surface. Follow-up OCT scans were conducted in weeks 4 and 8 post-treatment. Imaging parameters included 1000 A-scans and 100 B-scans per frame, covering a 3.2 mm × 3.2 mm area of the retina using a 6 mm retinal imaging lens [44]. The optic nerve served as the center of the scans. Total and different individual retinal layer thicknesses were determined by the Bioptigen segmentation software InVivoVue Diver (Leica Microsystem, Morrisville, NC, USA) at multiple cross-sectional images. The OriginPro 2018 (OriginLab Corporation, Northampton, MA, USA) statistical package was used for the statistical analyses.

### 2.7. Analysis of Ganglion Cell Changes on Whole-Mount Retinal Preparations

All rats (*n* = 53) were processed 8 weeks after microbead injection. The eyes were isolated, the corneas removed, and retinal tissue-containing ocular cups were fixed in 4% paraformaldehyde dissolved in 0.1 M PBS. After fixation, samples were washed in 0.1 M PBS for 1 h and dehydrated in ascending alcohol concentrations. To block non-specific binding, the samples for retinal whole mounts (*n* = 22) were incubated in a blocking solution of 5% normal donkey serum and 3% bovine serum in 0.3% PBST (phosphate buffer saline with Tween™ detergent) for 1 h. Retinas were then incubated overnight at 4 °C with the primary mouse anti-Brn3a (brain-specific homeobox/POU domain protein 3A) antibody (Sigma-Aldrich, Budapest, Hungary) diluted in 0.1 M PBS. Immunoreactivity was visualized using an Alexa Fluor 594 donkey anti-mouse secondary antibody (1:400, Jackson ImmunoResearch, Newmarket, UK), applied for 1 h. Retinal whole mounts were mounted on glass slides and coverslipped using Fluoroshield (Sigma-Aldrich, Budapest, Hungary). Images were captured using a Nikon Eclipse 80i epifluorescence microscope Auro-Science Consulting Ltd., Budapest, Hungary). Brn3a-positive retinal ganglion cells were quantified using ImageJ (version 1.54g, NIH, Bethesda, MD, USA) in three times eight regions per retina (four central and four peripheral regions, starting clockwise from 9 o’clock). Cells were counted within 500 × 500 µm^2^ fields and the results were compared with the existing literature. Images underwent contrast adjustments and labeling using Adobe Photoshop CS6 (Adobe Systems, Inc., San Jose, CA, USA).

### 2.8. Isolectin-B4 Staining on Retinal Vessels

Retinas (*n* = 22) were fixed as described in the “Analysis of ganglion cell changes on whole-mount retinal preparations” section, and the entire retinal tissue was isolated. Endothelial cells were labeled with biotinylated *Griffonia simplicifolia (DC.) Baill.* (Fabaceae) Isolectin-B4 (Isolectin GS-IB4 from *Griffonia simplicifolia*, Alexa Fluor 568 conjugate; Thermo Fischer Scientific, Waltham, MA, USA) as an endothelial-specific marker. The samples were incubated overnight at 4 °C. The retinas were spread on glass slides (Thermo Fischer Scientific, Waltham, MA, USA) and coverslipped. Images of the preparations were captured using a Nikon Eclipse 80i epifluorescence microscope (4× magnification, approximately 2500 × 2500 area, 150 µm/pixel). Images were processed using Adobe Photoshop CS6 (Adobe Systems, Inc., San Jose, CA, USA) with adjustments limited to contrast modifications and arrangement of labels. Vascular density was quantified using the ImageJ Vessel Analysis plugin (version 1.53, National Institutes of Health, Bethesda, MD, USA), which calculates the ratio of vessel area to total area and measures vessel diameters in pre-processed multi-channel images. The plugin generates a binary image for density calculation and provides tools for diameter measurement.

### 2.9. Western Blot

For Western blot analysis, retinal tissue homogenates from four animals were pooled into one Eppendorf tube, with a total of 26 animals used across all groups. Samples were stored at −80 °C until processing. Frozen tissues were homogenized using an Ultra-Turrax and Potter homogenizer in 500 μL lysis buffer containing 50 mM Tris, 50 mM EDTA (ethylenediaminetetraacetic acid), 0.5% protease inhibitor cocktail (Sigma-Aldrich, Budapest, Hungary), and 0.5% phosphatase inhibitor cocktail (Sigma-Aldrich, Budapest, Hungary; pH = 7.4). Homogenates were sonicated and protein concentrations were determined using the DC™ Protein Assay kit (Bio-Rad Laboratories, Hercules, CA, USA). Tissue lysates were diluted with Laemmli buffer, boiled for 5 min, centrifuged at 13,300 rpm for 10 min, and the supernatants were collected for further analysis. Proteins (20 μg per lane) were separated on SDS-PAGE (sodium dodecyl-sulfate polyacrylamide gel electrophoresis) gels and transferred to nitrocellulose membranes. Membranes were blocked in Tris-buffered saline (TBS) with 0.1% Tween-20 (Cell Signaling Technology, Danvers, MA, USA) and incubated overnight at 4 °C with primary antibodies, including anti-GAPDH (glyceraldehyde-3-phosphate dehydrogenase; 1:20,000; Millipore, Budapest, Hungary), anti-NFκB (1:500; Abcam, Cambridge, UK), anti-GFAP (glial fibrillary acidic protein; 1:1000; Sigma-Aldrich, Budapest, Hungary), anti-Bax (Bcl-2 associated X-protein; 1:500; Abcam, Cambridge, UK), anti-BDNF (brain-derived neurotrophic factor; 1:1000; Abcam, UK), anti-CREB (cAMP response element-binding protein; 1:1000; Cell Signaling Technology, Danvers, MA, USA), and anti-HIF1α (Hypoxia-Inducible Factor 1-alpha; 1:1000; Sigma-Aldrich, Budapest, Hungary). Membranes were washed six times with 0.2% Tween-20 in TBS (pH = 7.5) and incubated with horseradish peroxidase-conjugated secondary antibodies (1:3000; Bio-Rad, Budapest, Hungary; 1:2000; Cell Signaling Technology, Danvers, MA, USA). Bands were visualized using enhanced chemiluminescence with the Pierce ECL Western Blotting Substrate (Thermo Fisher Scientific, Waltham, MA, USA). Experiments were repeated three times, and the results were averaged. Band intensities were quantified using ImageJ software (version 1.54g) and normalized to GAPDH as an internal control. Data are represented as arbitrary units of pixel density.

### 2.10. Statistical Analysis

The collected data were analyzed using normality tests followed by ANOVAs with Fischer post hoc tests (OriginPro 2018). In the context of ERG, we applied a two-sample t-test with Welch’s correction for the F-test. Results were graphically represented using GraphPad Prism 5. Data are presented as “mean ± standard error of mean (SEM)” with differences considered significant at *p* < 0.05.

## 3. Results

### 3.1. Properties of Herbal Eye Drops

During the eight-week experiment, Sprague Dawley rats did not exhibit any allergic symptoms (e.g., itching, conjunctivitis, etc.), complications (e.g., chemical eye injury, corneal ulcer and perforation, periorbital epithelial deficiency due to itching and scratching, etc.), or side effects (e.g., increased IOP, corneal opacity, corneal precipitates, etc.) to either the combined herbal active ingredient eye drops or the placebo Hyabak^®^ artificial tears. The cornea, conjunctiva, and periocular regions of rats treated with the combined eye drops remained intact until the end of the experiment. In collaboration with the Institute of Medical Chemistry at the Faculty of Medicine, University of Szeged (Gabor Toth M.D., Ph.D. and his colleagues), a pharmacotechnology analysis was conducted to assess the stability of the eye drops at room temperature. Based on the HPLC (high-performance liquid chromatography) results provided, the metabolic rate of the product was 5% on day 10 and exceeded 20% after 14 days, indicating that the herbal eye drops are stable at room temperature.These stability results correlate with the study by Razboršek et al. on rosmarinic acid, which documented an 18–25% metabolism rate during one month of refrigerator storage [50]. In our experiments, the active ingredient was stored in an isolated +4 °C laboratory refrigerator for the entire 8 weeks, with fresh eye drops replenished weekly. Based on our observations, the application of the triple herbal combination eye drops provides the most optimal results in avoiding side effects and complications. A significant advantage of the formulation is its stability at room temperature, but according to our results and previous studies, its bioactivity can be preserved longer when stored in a refrigerator.

### 3.2. Effects of Combined Herbal Eye Drops on IOP

**Figure 1:**
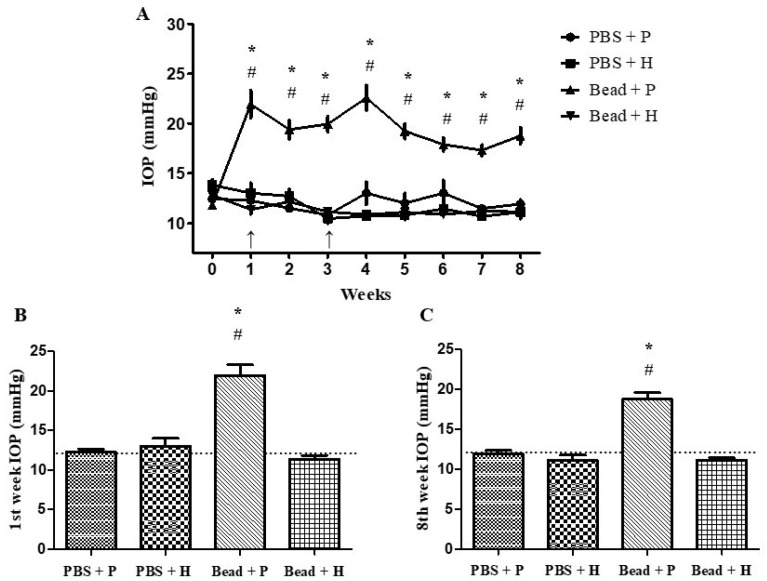
(**A**–**C**) Changes in IOP in Sprague Dawley rats during the eight experimental weeks. Comparison of IOP values in week 1 (**B**) and week 8 (**C**). Intracamerally applied microbeads significantly increased IOP. (*) *p* < 0.05 vs. PBS + P; (#) *p* < 0.05 vs. Bead + H. The time of microbead administration is indicated by an arrow (↑) in the (**A**) graph. Baseline is indicated as dotted lines in (**B**,**C**) graphs. Values are expressed in mean ± standard error of the mean (SEM). Abbreviations: PBS + P—control group; PBS + H—herbal treatment control; Bead + P—placebo-treated glaucoma group; Bead + H—herbal-treated glaucoma group; IOP—intraocular pressure.

In our ocular hypertension animal model (mimicking primary acute-angle closure or secondary open-angle glaucoma) by injecting 10 µm polystyrene microbeads into the anterior chamber, obstructing the trabecular meshwork and reducing or halting aqueous humor outflow. The increase in IOP, similar to that observed in human patients, exacerbates retinal and optic nerve damage over time [3]. Our IOP measurements align with values from the previous literature [44,51]. Following microbead implantation, the Bead + P group showed 21.95 ± 1.37 mmHg in week 1 and 22.59 ± 1.26 mmHg in week 4 after the second bead injection. Initial IOP values did not differ significantly among groups (Figure 1A). A significant IOP difference (*p* < 0.001) emerged in week 1 between the placebo-treated glaucoma group (Bead + P) and the combined agent-treated glaucoma group (Bead + H) (Figure 1A–C). This trend persisted throughout the 8 weeks (Figure 1A), with detailed weekly percentage changes in IOP values provided in Table 2. The differences between Bead + P and the other three groups were statistically significant (PBS + P: *p* < 0.001, PBS + H: *p* < 0.001, Bead + H: *p* < 0.001) from week 1 onwards (Figure 1A–C and Table 2). IOP peaks were observed after microbead injections in weeks 1 and 4 (Figure 1A and Table 2), confirming the effectiveness of our glaucoma model. IOP levels in PBS + P, PBS + H, and Bead + H groups remained near baseline over the 8 weeks (Figure 1A), indicating that our combined agent is beneficial for IOP-dependent glaucoma pathomechanisms without adverse effects on healthy eyes (Figure 1A–C and Table 2). Compared to the control group (PBS + P), the Bead + P group developed IOP values characteristic of secondary open-angle glaucoma (*p* < 0.001), showing no therapeutic response to placebo artificial tears (Figure 1A–C and Table 2).

### 3.3. Effects of Herbal Eye Drop Treatment on Retinal Ganglion Cells (RGCs) and Their Immunohistochemical (Brn3a WholeMount) Expression

**Figure 2:**
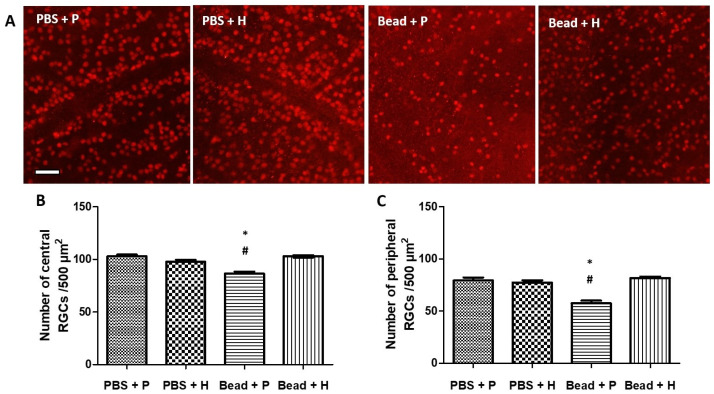
Quantitative morphometric analysis of RGCs. Representative whole-mounts stained by Brn3a in the four examined groups (**A**). Glaucomatous damage was mainly evident as a significant reduction in the number of cells between the GCL of the central (**B**) and peripheral (**C**) retinal area in the Bead + P group compared to the PBS + P and Bead + H groups: Bead + P: (central 86.4 ± 2.0 cells/500 µm^2^; *p* < 0.05; and peripheric 57.58 cells ± 2.43/500 µm^2^; *p* < 0.05). No significant differences were found between the control groups and the glaucoma group treated with the combined herbal compound. (*) *p* < 0.05 vs. PBS + P; (#) *p* < 0.05 vs. Bead + H. Values are expressed in mean ± standard error of the mean (SEM). Abbreviations: RGCs—retinal ganglion cells; PBS + P—control group; PBS + H—herbal treatment control; Bead + P—placebo-treated glaucoma group; Bead + H—herbal treatment glaucoma group. Scale bar—100 µm.

Rats typically have around 100,000 (86,282–97,609) ganglion cells per retina [52,53,54], while humans show greater variability (0.7–1.5 million) [55]. Reduced Brn3a expression is observed before RGC death, making it a useful marker for studying RGC loss and neuroprotective treatments [56]. In our immunofluorescent microscopy images, the control groups (PBS + P and PBS + H) showed no significant changes in Brn3a expression in RGCs (Figure 2A). The combined herbal treatment group (Bead + H) showed slight Brn3a reduction but maintained similar histological integrity to the control groups (Figure 2A). The quantitative analysis revealed an increase in RGC survival in the treated group compared to the controls. Specifically, the treated group showed a 16% and 27% higher survival rate of RGCs in the central and peripheric regions, respectively, indicating the neuroprotective effects of the administration (Table 3). The use of Brn3a markers allowed for accurate quantification of RGC survival, addressing the authors’ concerns about the need for more comprehensive evaluation.

Quantitative analysis of surviving RGCs in whole-mount retina samples confirmed no significant difference between the control groups (PBS + P: central 102.97 ± 1.55 cells/500 µm^2^, peripheric 79.36 ± 2.87 cells/500 µm^2^; PBS + H: central 97.83 ± 1.88 cells/500 µm^2^, peripheric 77.30 ± 2.23 cells/500 µm^2^) and the combined treatment glaucoma group (Bead + H: central 102.93 ± 1.08 cells/500 µm^2^, peripheric 80.41 ± 1.5 cells/500 µm^2^) (Figure 2B,C). In contrast, the placebo-treated glaucoma group (Bead + P) showed a significant decrease in Brn3a-positive RGCs (central 86.4 ± 2.0 cells/500 µm^2^; PBS + P: *p* < 0.001, PBS + H: *p* < 0.001; and peripheric 57.58 ± 2.43 cells/500 µm^2^; PBS + P: *p* < 0.001, PBS + H: *p* < 0.001) compared to the controls (Figure 2B,C). The combined treatment group had Brn3a expression levels similar to the controls (Figure 2B,C).

### 3.4. Effects of Herbal Eye Drops Treatment on Retinal Vessels (Isolectin-B4 Expression)

**Figure 3:**
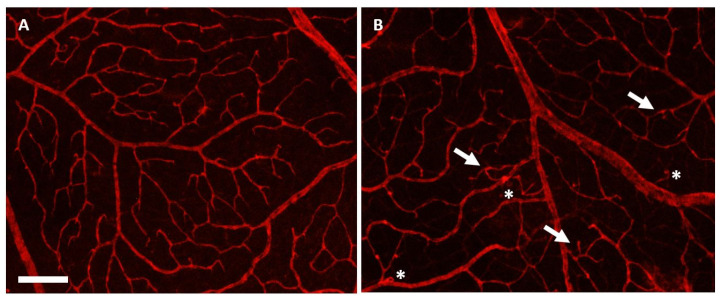
Retina whole mount with an endothelial cell-specific marker (Isolectin-B4). Images of retina preparations were taken at 40× microscopic magnification (scale bar—500 µm). In the control group (PBS + P) (**A**), the condition of the retinal vessels appears macroscopically intact, reflecting a normal state. In the glaucoma placebo-treated group (Bead + P) (**B**), microaneurysms (indicated by asterisks) and new anastomoses caused by neovascularization (indicated by arrows) are observed, indicating retinal ischemia and damage. Faintly visible are the anastomoses in the deeper layers of the glaucomatous retina (**B**), which are absent in the healthy specimen (**A**).

Immunohistochemical staining with the endothelial cell-specific marker, biotinylated *G. simplicifolia* Isolectin-B4, labels the retinal macro- and microvasculature by binding to the endothelial cells of the blood vessels [57,58]. In the control groups (PBS + P and PBS + H), the retinal vessels appeared macroscopically intact, reflecting a healthy condition (Figure 3A). However, in the glaucoma placebo-treated group (Bead + P), vascular injuries, microaneurysms, and microangiopathies were documented, indicating retinal ischemia and damage (Figure 3B).

**Figure 4:**
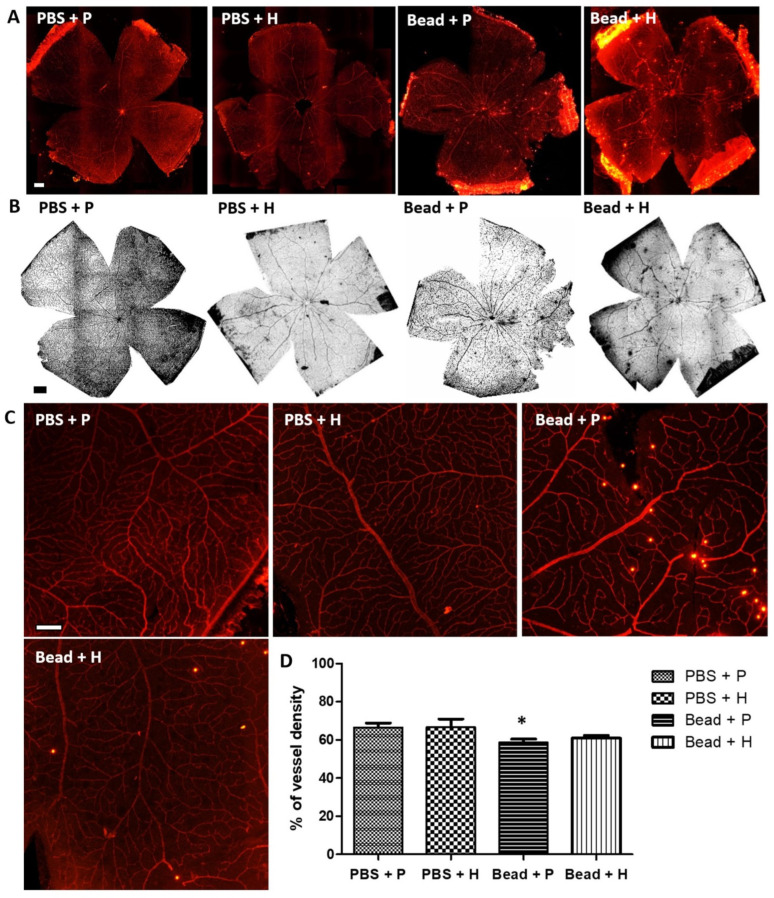
Retina whole mount with endothelial cell-specific marker (Isolectin-B4). Images of retina preparations were taken at 4× microscopic magnification (Series (**A**), scale bar–500 µm). The vascular condition of the retina was made contrast visible using the ImageJ (version 1.54g, NIH, USA) vessel density program plugin (Series (**B**), scale bar—500 µm). Magnified images (Series (**C**), magnification— 20×; scale bar—100 µm) were taken from a 500 µm^2^ peripheral area. In the percentage of vascularization of the retina samples, the placebo-treated glaucoma group (Bead + P: 58.73 ± 1.69%) was significantly (* *p* < 0.05) lower compared to the control group (PBS + P: 66.55 ± 2.36%) (**D**). The difference between PBS + P, PBS + H (66.74 ± 4.2%), and Bead + H (60.94 ± 1.47%) was not statistically significant. Values are expressed in mean ± standard error of the mean (SEM). Abbreviations: PBS + P—control group; PBS + H—herbal treatment control; Bead + P—placebo-treated glaucoma group; Bead + H—herbal treatment glaucoma group.

The retinal vasculature of the glaucoma animals treated with the combined herbal agent (Bead + H) showed similar morphology to that of the control groups (Figure 4A–C). Applying quantitative methods to analyze retinal vascular density, significant differences (*p* < 0.05) between our study groups could be detected comparing PBS + P (66.55 ± 2.36%) and Bead + P (58.73 ± 1.69%) (Figure 4D). Overall, microcirculatory changes in the retina and choroid lead to retinal damage from impaired circulation. Additional IOP fluctuations cause more harmful vascular dysregulation than stable suboptimal IOP, leading to RGC death and optic neuropathy progression [3,59].

### 3.5. Retinoprotective Effects of Herbal Eye Drop Treatment on Morphological Changes

**Figure 5:**
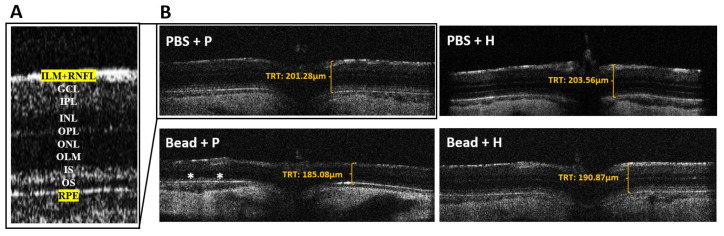
SD-OCT B-scan detailed retinal layers (**A**) and images (**B**) of Sprague Dawley rat retinas. The significantly thinner retinal structure of the glaucomatous rat group treated with placebo (Bead + P) can also be confirmed when compared with the OCT images, highlighting the total retinal thickness (TRT). The regions marked with an asterisk (*) on the diagram indicate areas where the retinal cross-section has significantly thinned. Abbreviations: PBS + P— control group; PBS + H—herbal treatment control; Bead + P—placebo-treated glaucoma group; Bead + H—herbal treatment glaucoma group; ILM—inner limiting membrane; RNFL—retinal nerve fiber layer; GCL—ganglion cell layer; IPL—inner plexiform layer; INL—inner nuclear layer; OPL—outer plexiform layer; ONL—outer nuclear layer; OLM—outer limiting membrane; IS—(photoreceptor’s) inner segment; OS—(photoreceptor’s) outer segment; RPE—retinal pigment epithelium; TRT—total retinal thickness.

Using OCT measurements, we found that the retinal layers of glaucomatous placebo-treated (Bead + P) animals showed signs of severe degeneration compared to the control groups (Figure 5A,B).

**Figure 6:**
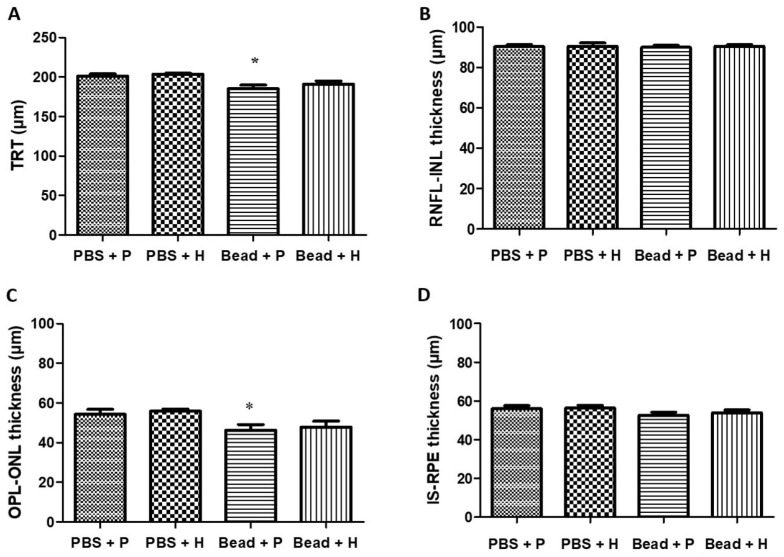
Comparison of total retinal thickness (TRT) (**A**) and different retinal layer thicknesses in the 8th experimental week (**B**) from the retinal nerve fiber layer (RNFL) to the inner nuclear layer (INL); (**C**) from the outer plexiform layer (OPL) to the outer nuclear layer (ONL); (**D**) from the inner segment of the photoreceptors (IS) to the retinal pigment epithelium (RPE) measured during OCT examination of Sprague Dawley rats with those of the combined herbal eye drop-treated groups; (*) *p* < 0.05 vs. PBS + P. Values are expressed in mean ± standard error of the mean (SEM). Abbreviations: PBS + P—control group; PBS + H—herbal treatment control; Bead + P—placebo-treated glaucoma group; Bead + H—herbal treatment glaucoma group.

Quantitative results were derived from reports generated by the OCT In Vivo Vue software (version 2.4) for further analysis. At the initial measurement (day 0 of the experiment), there were no significant differences in total retinal thickness, RNFL-INL, OPL-ONL, or IS-RPE layers among the groups (Table 4). By week 4, no significant differences were found between the control groups (PBS + P and PBS + H) and the herbal-treated glaucoma group (Bead + H). However, the placebo-treated glaucoma group (Bead + P) showed a documented decrease in total retinal thickness (Table 4). At the final (8th) week examination, the measured values for the PBS control groups (PBS + P: 201.28 ± 2.74 µm, and PBS + H: 203.56 ± 1.23 µm) and the treated glaucoma group (Bead + H: 190.87 ± 4.1 µm) did not show significant differences. In contrast, the Bead + P group exhibited significant retinal layer thinning (185.08 ± 4.75 µm; *p* = 0.01), similar to the week 4 data (Table 4 and Figure 6A–D). Our OCT measurements indicate that the retina in the glaucoma group (Bead + P) significantly changed compared to the other three groups (*p* = 0.01) due to retinal atrophy. The combined herbal treatment provided significant protection for the integrity of retinal layers in the Bead + H group.

### 3.6. Effect of Herbal Eye Drops on Visual Responses

**Figure 7:**
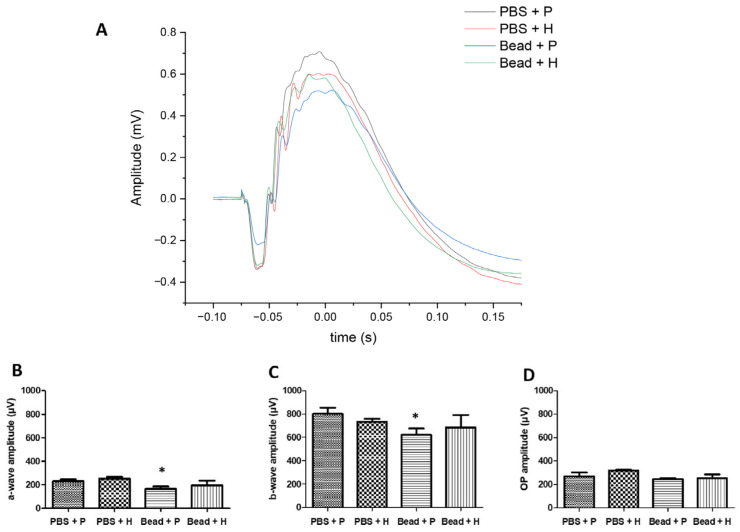
During ERG testing in Sprague Dawley rats, the average amplitudes of the a-waves, b-waves, and oscillatory potentials were recorded in the eighth experimental week (**B**–**D**). (*) *p* < 0.05 vs. PBS + P. The average waveforms (**A**) are consistent with the (**B**,**C**) graphs, with significant differences observed in the amplitude of the a- and b-waves (**A**–**C**). Values are expressed as mean ± standard error of the mean (SEM). Abbreviations: OP—oscillatory potential; PBS + P—control group; PBS + H—combined active treatment control group—Bead + P—placebo-treated glaucomatous group; Bead + H—combined active treatment glaucomatous group.

In full-field ERG analysis, scotopic a- and b-wave amplitudes in control eyes (PBS + P and PBS + H) were similar: PBS + P: a-wave = 230.79 ± 15.75 µV, b-wave = 801.13 ± 51.56 µV; and PBS + H: a-wave = 253.62 ± 13.66 µV, b-wave = 733.75 ± 23.42 µV (Figure 7A–C). In glaucomatous rats treated with the combined herbal formulation (Bead + H), ERG waveforms showed similar patterns to the control groups (PBS + P and PBS + H) (Figure 7A,B), showing significant functional protection. In the case of a two-sample t-test with unequal variances, based on the one-tailed *p* (T ≤ t) values, the amplitude values of the Bead + P group are significantly lower compared to the amplitude values of the PBS + P group. A significant reduction in a-wave amplitude was observed in the placebo-treated glaucomatous group (Bead + P: a-wave = 165.31 ± 19.9 µV) compared to the control group (PBS + P) (Figure 7A,B; *p* = 0.01). The b-wave also reached statistical significance (*p* = 0.02) in the Bead + P group (b-wave = 620.75 ± 54.2 µV) compared to PBS + P (Figure 7C). In terms of changes in the oscillatory potential waveform, the Bead + P group (212.29 ± 16.83 µV) did not show a significant difference compared to the PBS + P group (245.98 ± 8.1 µV) (Figure 7D, *p* = 0.26).

### 3.7. Western Blot Protein Analysis

**Figure 8:**
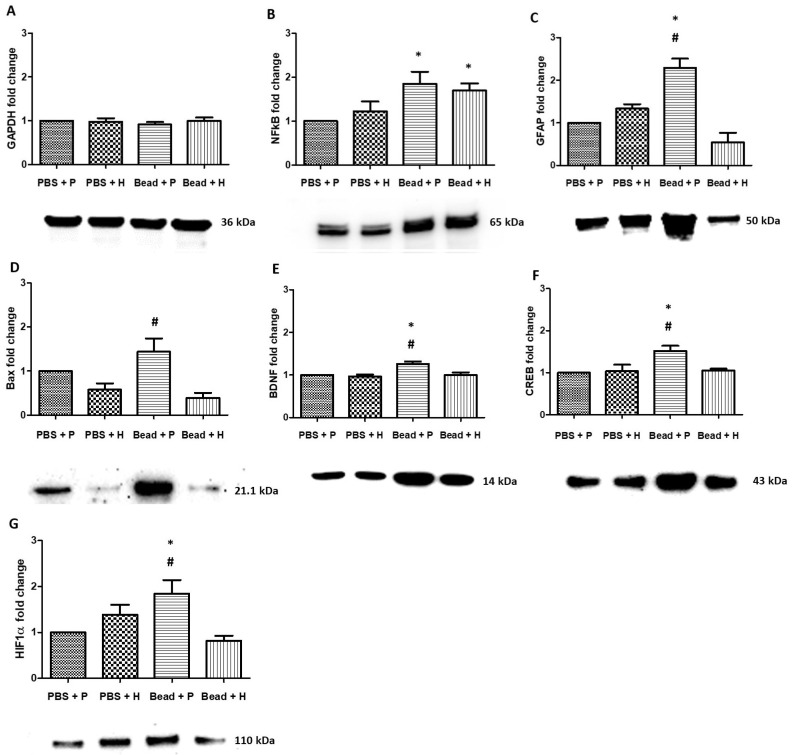
Western blot analysis of Sprague Dawley rat retina samples (**A**–**G**). GAPDH protein (**A**) was chosen for quantitative standardization of sample amounts and normalization of results. (*) *p* < 0.05 vs. PBS + P; (#) *p* < 0.05 vs. Bead + H. Values are expressed in mean ± standard error of mean (SEM). Abbreviations: PBS + P—control group; PBS + H—herbal treatment control; Bead + P—placebo-treated glaucoma group; Bead + H—herbal treatment glaucoma group; GAPDH—glyceraldehyde-3-phosphate dehydrogenase (**A**); NFκB—nuclear factor κ-B (**B**); GFAP—glial fibrillary acidic protein (**C**); Bax—Bcl-2 Associated X-protein (**D**); BDNF—brain-derived neurotrophic factor (**E**); CREB—cAMP response element-binding protein (**F**); HIF1α—hypoxia-inducible factor 1-alpha (**G**).

GAPDH protein was selected to quantitatively standardize sample quantity and normalize results. This has been used in previous studies [60] and is equally expressed in all samples (Figure 8A). Elevated IOP increased stress-induced cellular processes and apoptosis in the glaucoma groups (Bead + P and Bead + H), and both showed significant changes in NFκB protein compared to PBS + P (Figure 8B; *p* = 0.01, and *p* = 0.03). Placebo-treated glaucoma rats (Bead + P) showed significantly increased GFAP levels compared to the PBS + P and Bead + H groups (Figure 8C; *p* < 0.001, and *p* < 0.001). The GFAP levels in the Bead + H group were similar to those in the PBS + P and PBS + H control groups.

Our study demonstrated that combined herbal treatment (Bead + H) reduces the expression of Bax in glaucoma. The levels of Bax, a marker of neuronal cell death and apoptosis, were significantly higher in the placebo-treated glaucoma group (Bead + P) compared to the Bead + H group (Figure 8D, *p* = 0.001).

BDNF (brain-derived neurotrophic factor) protein expression significantly increased in the Bead + P group compared to PBS + P and Bead + H groups (Figure 8E, *p* = 0.007 and *p* = 0.006, respectively), which means more impaired neurotransmission leading to overcompensation and protein accumulation. CREB protein expression was significantly higher in placebo-treated glaucomatous rats (Bead + P) compared to the PBS + P control and Bead + H groups (Figure 8F, *p* = 0.006, and *p* = 0.01, respectively).

In the Bead + P group, the expression of retinal hypoxia marker (HIF1α) was significantly higher compared to the PBS + P and Bead + H groups (Figure 8G, *p* = 0.01, and *p* = 0.004). This indicates that our combined treatment (Bead + H) protected the retinas of glaucomatous rats from hypoxia-induced neovascularization (Figure 8G).

## 4. Discussion

### 4.1. Effects of Combined Herbal Eye Drops on IOP

Chronic elevation of IOP due to angle closure or increased trabecular meshwork resistance can cause ciliary body atrophy, reducing aqueous humor production and leading to IOP reduction [61]. Elevated IOP over weeks poses a 10–19% increased risk per mmHg for progression according to human data [62]. This supports the efficacy of our combined formulation, as the Bead + H-treated group maintained baseline average IOP values compared to the control group (PBS + P) over the 8-week period (Figure 1). Szabo et al. also successfully induced and maintained IOP elevation in glaucoma groups over 8 weeks [44]. Our study confirmed the expected IOP-lowering effect of our herbal combination, although its precise pharmacological mechanism—based on previous studies—remains unknown. The IOP reduction achieved with our herbal combination may benefit patients with glaucoma. Amato et al. conducted a similar glaucoma animal model study using oral *Mentha spicata* extract; however, it did not alter IOP, despite demonstrating positive neuroprotective retinal effects. Limitations include non-topical application and lack of combination with other herbs that might reveal IOP-dependent mechanisms [63]. Based on the hypothesis of the authors and the findings of Agarwal et al., *F. vulgare* may possess inherent IOP-lowering effects potentially through the non-pigmented cells of the ciliary body (similar to β-blockers such as timolol and betaxolol) or by improving uveoscleral outflow (similar to prostaglandin analogs such as latanoprost, bimatoprost, and tafluprost). However, with current methodologies, the exact mechanism remains unknown. The additive IOP-lowering effect of *R. officinalis* and *H. italicum* could be explained by an anti-inflammatory mechanism similar to steroids. Topical dexamethasone is frequently used in the clinical management of acute primary angle closure attacks, and studies have demonstrated the IOP-lowering effects of steroid-based anti-inflammatory therapy in both humans and rats [64,65]. Steroids exert their action by inhibiting the NFκB and COX inflammatory pathways, highlighting the possible synergism between *R. officinalis* and *H. italicum*, as no comparable independent molecular mechanism has been identified for *F. vulgare* [11,12,13,20]. Approximately 10% of the population are known to be steroid responders, meaning they experience painless extreme IOP spikes in response to periocular steroid therapies [66]. Furthermore, prolonged steroid use can lead to secondary open-angle glaucoma through trabecular meshwork remodeling [66]. Therefore, the developed anti-inflammatory herbal combination could represent a promising alternative for steroid responders and patients with complex forms of glaucoma.

### 4.2. Effects of Herbal Eye Drop Treatment on Histological Changes of the Retina

Szabo et al. reported significant retinal degeneration between the ILM-OLM layers in glaucoma rats, indicating disease progression. Their treatment with PACAP 1-38 (pituitary adenylate cyclase-activating polypeptide 1-38) demonstrated retinal protection, comparable to the protective effects observed with our combined herbal treatment across all retinal layers [44,48,67]. The literature suggests that in glaucoma both retinal ganglion cells and the entire retinal vertical pathway, including photoreceptors, are compromised [44,48,67]. The OCT analysis in a glaucoma rat model of Guo et al. revealed a strong correlation between disease progression and the thinning of the total retinal thickness, RNFL, and ONL layers, attributed to cumulative IOP effects [68]. This trend was also evident in our study, particularly following the extreme IOP elevation after the second microbead injection.

### 4.3. Effects of Herbal Eye Drop Treatment on Retinal Ganglion Cells (RGCs) and Their Immunohistochemical (Brn3a Whole-Mount) Expression

Reduced Brn3a expression is detected prior to RGC death, serving as a useful marker for investigating RGC loss and evaluating neuroprotective treatments [56]. Our results indicate that the combined herbal eye drop treatment is protective for RGC integrity in glaucoma. Similar to our findings, Szabo et al. reported a significant decrease in RGCs in their placebo-treated glaucoma group (Bead + P) compared to PACAP 1-38 eye drop-treated and control groups [44].

### 4.4. Effect of Herbal Eye Drops on Visual Responses

Increased IOP exerts significant mechanical stress on retinal tissues, disrupting retinal circulation in relation to arterial pressure. Similar to scotopic and photopic a-waves in mammals, the rat scotopic a-wave involves hyperpolarizing bipolar cells. Despite a reduced a-wave, the photopic b-wave in rats is nearly 60% larger than the corresponding scotopic b-wave, reflecting the relatively high number of cone bipolar cells in the rat retina [69]. Although the literature typically identifies the b-wave as an electrophysiological marker sensitive to IOP changes [69], our findings did not align with this in our experimental model. Previous studies by Bayer et al. showed a 40% decrease in a-waves after 40 days in glaucomatous rats, while b-waves decreased by only 15%. However, both a- and b-waves significantly decreased with prolonged implicit times [48]. Guo et al. confirmed in their study that photoreceptor damage significantly reduces scotopic a-waves [68], which was similarly observed in our results (Figure 7A,B). The oral formulation of *M. spicata* extract developed by Amato et al. did not produce significant changes in either the photopic or scotopic ERG in any rat group, thereby demonstrating dose-dependent neuroprotection, although it was not tested as a topical formulation [63].

### 4.5. Properties of Herbal Eye Drops by Western Blot Protein Analysis

Retinal neuronal viability relies on precise homeostatic processes and signaling pathways. However, its intricate structure and high metabolic demand make the retina susceptible to various stressors. When these stress levels exceed a critical threshold, protective mechanisms are activated. In particular, metabolic stressors can profoundly affect the retina by triggering pathways such as oxidative stress, neuroinflammation, excitotoxicity, mitochondrial damage, autophagy, and apoptosis [3]. Western blot detects specific proteins to explore these pathways. Phytopharmaceuticals can induce anti-apoptotic pathways and inhibit pro-apoptotic pathways, preventing further cellular and tissue damage [3]. Our study aimed to confirm the possible pathways and mechanisms of our combined herbal eye drop product.

NFκB plays a crucial role in supporting neuronal survival against inflammatory processes and is activated by stimuli such as hyperglycemia or hypoxia. In aging retinas, its expression increases, leading to the activation of Müller cells and microglia under stress. In glaucoma, elevated NFκB expression is observed in the GCL and INL [70,71]. Amato et al. also detected NFκB expression during their examination of oral *M. spicata* supplementation [63]. Elevated IOP further induces cellular stress in retinal astrocytes and glial cells, detectable by GFAP. Elevated GFAP is a marker of metabolic stress in retinal tissue, indicating a compensatory response for the survival of Müller cells and astrocyte glial cells [44,72,73]. Szabo et al. also demonstrated neuroprotective effects using GFAP and Brn3a in their rat model treated with PACAP 1-38 polypeptide [44]. The caspase cascade and Bcl-2/Bax pathways are crucial in intrinsic apoptotic processes, with Bax promoting mitochondrial membrane permeability and cytochrome C release, leading to apoptosis [74]. Pre-pro-BDNF and then cleaved-pro-BDNF precursor, stored in dendrites or axons, is released in both pro- and mature BDNF forms, binding to TrkB (tropomyosin receptor kinase B) receptors to promote cell survival and neuroplasticity [3,75]. Amato et al. demonstrated the anti-apoptotic and neuroprotective effects of *M. spicata* extract in a glaucoma rat model through BDNF detection [63]. CREB activity in neurons is linked to processes like proliferation, differentiation, survival, synaptic potentiation, neurogenesis, and plasticity. Activated CREB promotes BDNF expressions, and vice versa, through TrkB receptors. CREB inhibition triggers apoptosis, while chronic activation leads to neuron loss via excitotoxicity [76]. These findings suggest different types of MAPK-CREB pathway activation were observed in glial cells and RGCs [77]. CREB also mediates hypoxia-induced gene expression, including VEGF-A, contributing to pathological retinal neovascularization [78]. HIF1α mediates the retinal hypoxia pathway, initiating neovascularization through VEGF. Immunohistochemical analysis confirmed elevated HIF1α levels in post-mortem glaucomatous retinal tissue, indicating hypoxic conditions in vessel endothelial cells, RPE, RGCs, Müller cells, pericytes, and other neurons [3,59].

Szabo et al. and Patko et al. conducted experiments using methodologies similar to those employed in the current rodent glaucoma model. However, there are notable differences between their studies and the present work. While the former studies focused on the neuroprotective effects of PACAP [44,59], the present study investigated the combination of three herbal active ingredients in a glaucomatous rat model. Although group formations and the 8-week experimental period were comparable across studies, the treatment protocols differed; Szabo and Patko et al. administered eye drops three times daily, whereas the current study applied treatments only twice daily [44,59]. Differences in scientific methodology also exist. For instance, the present study conducted OCT examinations at weeks 1, 4, and 8, while the earlier studies only performed assessments at the beginning and end of the experimental period [44,59]. Szabo et al. performed histology, immunohistochemistry, Brn3a whole-mount retina analysis, and OCT to assess morphological and morphometric changes in the retina, but did not perform Western blot [44]. Patko et al. focused on the effects of PACAP on retinal circulation. In addition to the basic OCT examination, they assessed retinal neovascularization using VEGF and HIF1α immunohistochemistry and Western blot methods, as well as software-based assessments of retinal vascular structure. However, they did not include detailed ERG data in their findings [59]. In contrast, the current study on combined herbal eye drops focused on retinal vessel density, whereas Patko et al. also evaluated vessel lacunarity [59]. A key advantage of our study is the integration of multiple methodological approaches, along with the extension of Western blot analysis to several critical markers involved in anti-inflammatory, anti-apoptotic, and anti-neovascularization pathways.

**Figure 9:**
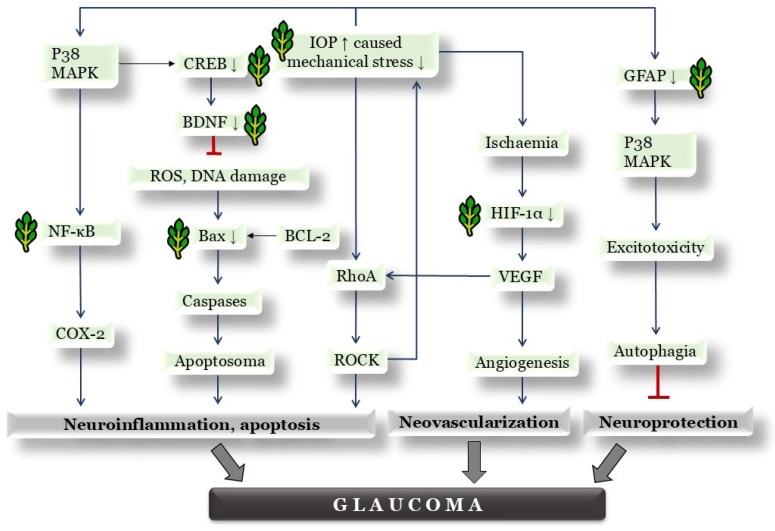
Main signaling downstream pathways of the three major retinal diseases in the self-edited figure. Our complex herbal active ingredient exerts its anti-glaucoma effect through the molecular pathways we investigated, indicated by the “⸙” symbol; red color indicates inhibitory processes. The downward arrows (↓) indicate a reduction in protein expression as a result of the combined herbal treatment. Abbreviations: IOP↑—increase of intraocular pressure; NFκB—nuclear factor κ-B; Bax—Bcl-2 Associated X-protein; BCL-2—B cell lymphoma 2; BDNF—brain-derived neurotrophic factor; CREB—cAMP response element-binding protein; COX-2—cyclooxygenase-2; DNA—deoxyribonucleic acid; GFAP—glial fibrillary acidic protein; HIF1α—hypoxia-inducible factor 1-alpha; p38-MAPK—p38-mitogen-activated protein kinases; RhoA—Ras homolog family member A; ROCK—Rho associated coiled-coil forming kinase; ROS—reactive oxygen species; VEGF—vascular endothelial growth factor.

Overall, the development of the glaucoma model in the Bead + P and Bead + H groups can be considered successful. Retinal stress responses induced by elevated IOP and the activation of hypoxia-induced signaling pathways were initiated. These data suggest that our complex herbal formulation exerts anti-apoptotic effects and reduces neuronal inflammation, thereby providing neuroprotective effects. An additional advantage is the inhibition of angiogenesis. A previous literature review of the co-author illustrates a schematic diagram of the shared molecular pathways in glaucoma, age-related macular degeneration (AMD), and diabetic retinopathy [3]. Based on this, the simplified schematic in Figure 9 highlights the molecular pathways affected by our complex herbal preparation, supporting its potential application in glaucoma therapy.

## 5. Conclusions

Our objective was to develop an effective eye drop formulation that combines well-known herbal ingredients. We hypothesized that this formulation could lower IOP, reduce inflammation and oxidative stress, and provide neuroprotection, potentially slowing the progression of irreversible optic neuropathy, as supported by contemporary glaucoma research. A limitation of this study is the short experimental duration and the use of a single rodent model. Future research would benefit from longer-term studies and the translation of these findings to human glaucoma patient populations. In conclusion, our functional, structural, and molecular analyses indicate that the combined herbal formulation exerts potent protective effects against glaucoma development and demonstrates significant therapeutic potential as an eye drop treatment.

## Figures and Tables

**Table 1:** The main active ingredient content (%) of the aqueous and oil extracts of the herbs studied in the experiment. ND (no data)—no precise literature data found.

Chemical Compounds	*Rosmarinus officinalis*	*Foeniculum vulgare*	*Helichrysum italicum*	References ^a^
***Aqueous extract***				
*Apigenin*	0.01–0.2%	0.1–12.5%	ND	[23,24]
*Carnosic acid* ^b^	7.5–17.3%	ND	ND	[24,25]
*Carnosol*	0.5–3.0%	ND	0.1–0.2%	[24,25]
*Chlorogenic acid* ^b^	0.5–2.0%	0.11–6.8%	3.38%	[23,24,25,26]
*Luteolin*	0.1–0.5%	0.1–0.3%	ND	[24]
*Rosmanol*	0.5–2.0%	ND	ND	[24]
*Rosmarinic acid* ^b^	10.0–84.0%	14.9–18.0%	4.54%	[23,24,26,27,28]
*Rutoside*	ND	0.01–0.3%	0.0–0.19%	[24,25]
***Oil extract***				
*Geraniol*	0.5–1.8%	ND	3.0–6.80%	[26,29,30,31,32,33]
*Limonene* ^b^	1.5–5.0%	2.41–11.45%	2.17–6.07%	[24,34,35,36]
*Linalool*	1.41–6.2.0%	ND	2.8–4.7%	[24,27,29,30,32,33,34,37,38,39,40,41,42]
Trans-anethole ^b^	0.1–3.45%	54.26–88.28%	ND	[23,38]

^a^ Detailed references for the sources can be found in the bibliography. ^b^ The main pharmacologically active compounds are potentially responsible for the effects of the herbal combination used in the experiment.

**Table 2:** Percentage (%) changes in IOP values relative to the weekly control group (PBS + P) over the 8 weeks of the combined eye drops experiment. The percentage increase in IOP values in the placebo-treated glaucoma group (Bead + P) compared to the control group (PBS + P) is highlighted in bold with an asterisk (*), indicating significant (*p* < 0.05 vs. PBS + P) IOP elevation, which appeared as early as the first week following anterior chamber microbead injection. Negative percentage values indicate a decrease in IOP expressed as a percentage compared to the weekly PBS + P group. Abbreviations: PBS + P—control group; PBS + H—herbal treatment control; Bead + P—placebo-treated glaucoma group; Bead + H—herbal treatment glaucoma group.

Week	PBS + P	PBS + H	Bead + P	Bead + H
0.	0	11.13	−5.13	2.96
1.	0	6.12	78.83 *	−7.32
2.	0	10.60	68.65 *	5.83
3.	0	−3.43	83.93 *	2.89
4.	0	−17.62	73.58 *	−16.49
5.	0	−10.07	60.13 *	−7.49
6.	0	−12.09	37.50 *	−16.49
7.	0	−6.88	51.06 *	−2.14
8.	0	−6.85	57.41 *	−6.63

**Table 3:** The percentage loss of central and peripheral retinal ganglion cells (RGCs) compared to the control group (PBS + P). Abbreviations: PBS + P—control group; PBS + H—herbal treatment control; Bead + P—placebo-treated glaucoma group; Bead + H—herbal treatment glaucoma group.

	PBS + P	PBS + H	Bead + P	Bead + H
Central	0	−4.991	−16.078	−0.034
Peripheric	0	−2.590	−27.441	2.853

**Table 4:** The percentage change in relative layer thickness of retinal histological layers measured by OCT between groups over the 8 weeks of the experiment was compared to the control group (PBS + P). This comparison indicates how the current thickness of the retinal layers changed as a percentage relative to the weekly thickness of the PBS + P group. Significant thinning of the layer thicknesses, which indicates retinal damage in the placebo-treated glaucoma group from week 4, is highlighted in bold. Abbreviations: PBS + P—control group; PBS + H—herbal treatment control; Bead + P—placebo-treated glaucoma group; Bead + H—herbal treatment glaucoma group; RNFL—retinal nerve fiber layer; OPL-ONL—retinal layers between outer plexiform layer and outer nuclear layer; IS-RPE—retinal layers between photoreceptors’ internal segment and retinal pigment epithelium.

	**Total Retinal Thickness (TRT) (%)**			
	PBS + P	PBS + H	Bead + P	Bead + H
Week 1	0	−2.12	−4.99	−0.92
Week 4	0	−1.07	−8.18	−3.39
Week 8	0	1.14	−8.05	−5.17
	**RNFL-INL thickness (%)**			
	PBS + P	PBS + H	Bead + P	Bead + H
Week 1	0	−3.19	0.20	1.67
Week 4	0	0.33	0.82	3.52
Week 8	0	0.19	0.83	0.31
	**OPL-ONL thickness (%)**			
	PBS + P	PBS + H	Bead + P	Bead + H
Week 1	0	1.85	−6.49	−5.97
Week 4	0	1.35	−9.66	−4.40
week 8	0	2.78	−14.98	−11.82
	**IS-RPE thickness (%)**			
	PBS + P	PBS + H	Bead + P	Bead + H
week 1	0	−0.04	−4.61	−0.35
week 4	0	0.06	−8.01	−3.80
week 8	0	0.73	−6.03	−3.74

## Data Availability

The original contributions presented in this study are included in the article and the Hungarian Patent Application (No P2500037); further inquiries can be directed to the corresponding author.

## Abbreviations

The following abbreviations are used in this manuscript:

AChE	acetylcholinesterase
AMD	age-related macular degeneration
BCL-2	B cell lymphoma 2
BDNF	brain-derived neurotrophic factor
Brn3a	brain-specific homeobox/POU domain protein 3A
COX-2	cyclooxigenase-2
DNA	deoxyribonucleic acid
EDTA	ethylenediaminetetraacetic acid
ERG	electroretinography
FoNo	Formulae normales (standard formulations in the Pharmacopoeia Hungarica)
GABA	γ-aminobutyric acid
GAPDH	glyceraldehyde-3-phosphate dehydrogenase
GCL	ganglion cell layer
GFAP	glial fibrillary acidic protein
Hif1α	hypoxia-inducible factor 1-alpha
HPLC	high-performance liquid chromatography
IL	interleukin
ILM	inner limiting membrane
INL	internal nuclear layer
IOP	intraocular pressure
IPL	internal plexiform layer
IS	(photoreceptor) internal segment
ND	no data
NFκB	nuclear factor kappa B
OCT	optical coherence tomography
OLM	outer limiting membrane
ONL	outer nuclear layer
OPL	outer plexiform layer
OS	(photoreceptor) outer segment
PACAP	pituitary adenylate cyclase-activating polypeptide
PBS	phosphate buffer saline
PBST	phosphate buffer saline with Tween™ detergent
POAG	primary open-angle glaucoma
p38-MAPK	p38-mitogen-activated protein kinases
RGC	retinal ganglion cell
RhoA	Ras homolog family member A
RNFL	retinal nerve fiber layer
RPE	retinal pigment epithelium
ROCK	Rho associated coiled coil forming kinase
ROS	reactive oxygen species
SDS-PAGE	sodium dodecyl-sulfate polyacrylamide gel electrophoresis
SEM	standard error of mean
SD-OCT	spectral domain optical coherence tomography
TBS	tris-buffered saline
TGFβ	transforming growth factor beta
TNFα	tumor necrosis factor alpha
TRT	total retinal thickness
TrkB	tropomyosin receptor kinase B
VEGF	vascular endothelial growth factor
